# Comfort from suicidal cognition in recurrently depressed patients^☆^

**DOI:** 10.1016/j.jad.2013.11.006

**Published:** 2014-02

**Authors:** Catherine Crane, Thorsten Barnhofer, Danielle S. Duggan, Catrin Eames, Silvia Hepburn, Dhruvi Shah, J.Mark G. Williams

**Affiliations:** a Department of Psychiatry, University of Oxford, Warneford Hospital, Oxford OX3 7JX, UK; b Centre for Mindfulness Research and Practice, Bangor University, Bangor, UK; c Department of Palliative Care, Royal Free Hospital, Royal Free London NHS Foundation Trust, Pond Street, London NW3 2QG, UK

**Keywords:** Suicidality, Depression, Cognition, Comfort

## Abstract

**Background:**

Previous research has suggested that some individuals may obtain comfort from their suicidal cognitions.

**Method:**

This study explored clinical variables associated with comfort from suicidal cognition using a newly developed 5 item measure in 217 patients with a history of recurrent depression and suicidality, of whom 98 were followed up to at least one relapse to depression and reported data on suicidal ideation during the follow-up phase.

**Results:**

Results indicated that a minority of patients, around 15%, reported experiencing comfort from suicidal cognitions and that comfort was associated with several markers of a more severe clinical profile including both worst ever prior suicidal ideation and worst suicidal ideation over a 12 month follow-up period.

**Limitations:**

Few patients self-harmed during the follow-up period preventing an examination of associations between comfort and repetition of self-harm.

**Conclusions:**

These results, although preliminary, suggest that future theoretical and clinical research would benefit from further consideration of the concept of comfort from suicidal thinking.

## 1 Introduction

Suicidal ideation and suicidal behaviour are common but serious features of recurrent major depression. Once suicidal ideation has occurred during one episode of major depression it is more likely to occur again in a future episode than any of the other non-core symptoms (Williams et al., 2006), and it is known that information about a person's worst ever episode of suicidal ideation has more predictive validity for future suicide than their level of suicidal ideation at a given assessment point (Beck et al., 1999). Considerable attention has been directed towards understanding motivations and intentions for suicide and content of suicidal cognition. This research suggests that suicidal individuals experience profound feelings of entrapment (Williams et al., 2005) as well as hopelessness, burdensomeness and unlovability (Van Orden et al., 2010; Beck et al., 1990), and that suicidal behaviour may have both intrapersonal and interpersonal components (e.g. escaping distress versus communicating distress to others, e.g. May and Klonsky (2013)). Attention has also been directed towards the *form* that suicidal cognitions take, suggesting that verbal thoughts and vivid mental imagery relating to suicide commonly co-occur (e.g. Holmes et al., 2007; Crane et al., 2012; Hales et al., 2011) both in individuals with mood disorders who have experienced suicidal crises and in those who have been depressed but not actively suicidal. Finally there is increasing interest in patients' relationships with and responses to the occurrence of suicidal cognitions (Williams et al., 2011) and the impact of reactions to suicidal cognitions on ongoing vulnerability. For example, Morrison and O'Connor (2008) systematic review of 11 studies found consistent evidence for a link between rumination and suicidality and Pettit et al. (2009) have demonstrated that suppression of suicidal thoughts is associated with increased severity of such thoughts, both concurrently and prospectively over a 4 weeks period. These findings suggest that developing an understanding of both the content of a patient's suicidal cognitions and of the way they relate to those cognitions may be critical to inform clinical management.

One type of relationship to suicidal cognition which has received relatively little attention in the literature is the extent to which individuals gain comfort or relief from their suicidal cognitions. Indeed, whilst many individuals report trying to suppress or avoid suicidal cognitions, and experience them as ‘unwanted’, ‘beyond their own will’ and ego dystonic (e.g. Bradvik and Berglund, 2011), clinical experience suggests that for some, suicidal cognition may have comforting properties and become compelling and preoccupying. For example, in a study of suicidal imagery in unipolar and bipolar patients Hales et al. (2011) report on the appraisals patients ascribe to their most vivid suicidal imagery. Whilst many of the appraisals were negative (e.g. ‘something must be badly wrong’; ‘[the imagery] makes me feel like I am going to die’), others reported that the suicidal imagery represented an escape route, or a release (e.g. ‘[the images mean that] I will be released from this, all these thoughts’, ‘[when I experienced the image] I wanted to take the tablets to find calm’), and mean ratings of imagery-related comfort and distress were similar across the sample as a whole. Indeed the three studies of suicidal imagery that have addressed issues of comfort have all found similar mean ratings of comfort and distress associated with suicidal imagery (Holmes et al., 2007; Crane et al., 2012; Hales et al., 2011). For example in Holmes et al. two thirds of participants rated their suicidal images as moderately comforting or greater, whilst in the sample of Crane et al., the mean level of comfort was 4.6 on a scale where 5 corresponded to moderately comforting. It is important to note that in this study there was a strong negative correlation between comfort and distress, suggesting that the two appraisals or emotional responses to suicidal imagery do not tend to coexist within a single patient, at least in relation to a particular image or episode. Finally confirming this aspect of the experience of some suicidal people, Vatne and Naden (2011) describe in a qualitative study of 10 patients that for their participants ‘*the thought of suicide can provide consolation and comfort to go on living*’.

Joiner's Interpersonal Psychological Theory of Suicide (IPTS; e.g. Van Orden et al., 2010), which deals primarily with the constellation of psychological processes that increase an individual's capacity to engage in lethal self-harm, has particular relevance when considering how suicidal cognitions may acquire comforting properties. Considering deliberate self-harm, the IPTS suggests that gradually, through the action of habituation and opponent processes, emotional reactions to deliberate self-harming behaviours change such that ‘what was originally a pain and/or fear inducing experience… may become less frightening *as well as a source of emotional relief*’ (Van Orden et al., 2010, pp. 587, italics added). We argue that there is value in considering whether the same phenomenon of habituation may also occur in relation to intrusive suicidal cognitions, and whether this may account for the observation that some individuals experience comfort from suicidal thoughts. If this is the case it is likely to be concerning, because it suggests that there may be an elaboration or escalation in suicidal ideation over time. Consistent with this suggestion, in the study of suicidal cognition in previously depressed patients discussed above, Crane et al. (2012) found that increased ratings of suicidal imagery-related comfort were strongly associated with increased severity of worst-ever suicidal ideation. However to date no study has examined whether taking comfort from suicidal thoughts predicts severity of future suicidal ideation after controlling for prior history.

The current study utilised a short scale, developed by the authors, to investigate participants' ratings of comfort associated with suicidal cognition. We administered this scale to a large sample of patients with recurrent depression and a history of suicidality. We examined (a) the extent to which ratings of comfort were correlated with (i) severity of *prior* suicidal ideation and (ii) occurrence of *prior* suicidal behaviour, and (b) whether ratings of comfort were correlated with the severity of *subsequent* suicidal ideation during a 1 year follow-up period. We hypothesised first that, consistent with the previous work, individuals who rated suicidal cognitions as more comforting would have higher levels of worst ever suicidal ideation at the baseline assessment, and second, that controlling for worst ever prior suicidal ideation, those who reported greater comfort from suicidal cognitions at a baseline assessment would report more severe suicidal ideation when they experienced a relapse to depression over a subsequent 12 months follow-up period.

## 2 Method

### 2.1 Recruitment

Participants were recruited to a large randomised controlled trial of psychological treatment for prevention of recurrent depression (the Staying Well after Depression Trial), (Williams et al., 2010) from primary care and mental health care services and community advertising at two sites: Oxford, England and Bangor, Wales. Inclusion criteria at baseline assessment were (a) age between 18 and 70 years; (b) history of at least three episodes of Major Depression meeting DSM-IV-TR criteria of which two must have occurred within the last 5 years, and one within the last 2 years; (c) remission for the previous 8 weeks; and (d) informed consent from participants and their primary care physicians. Participants were excluded if they had (a) a history of schizophrenia, schizo-affective disorder, bipolar disorder, current misuse of alcohol or other substance, organic mental disorder, pervasive developmental delay, primary diagnosis of obsessive-compulsive disorder or eating disorder, or regular non-suicidal self-injury; (b) inability to complete research assessments through difficulty with English, visual impairment, or cognitive difficulties and (c) if they were currently in psychotherapy more than once per month.

### 2.2 Ethics statement

This study was approved by Oxfordshire Research Ethics Committee C and North Wales Research Ethics Committee. The funder played no role in study design or conduct, or the decision to submit this paper for publication.

### 2.3 Procedure

Following screening for eligibility participants completed a baseline assessment including measures of current and prior suicidality, clinical history and the comfort from suicide measure, as well as a range of other measures not relevant to the current report (see Williams et al., 2010) and following treatment or an equivalent waiting period (approximately 8–12 weeks) participants were reassessed on the same measures. Participants were then followed up for 12 months with assessments at approximately 3 monthly intervals. At each assessment participants were interviewed to determine whether they had relapsed since the previous assessment, and also reported on current levels of suicidal ideation. For the purposes of the current study, data analysis focused on individuals who relapsed to major depression at least once during follow-up and who reported data on suicidal ideation at the assessment at which a relapse was recorded. There were no significant main effects or interactions with treatment group, and since all participants had experienced a relapse to depression (which the treatments were aiming to prevent) treatment is not considered further in this paper.

### 2.4 Measures

#### 2.4.1 Comfort from suicidal thoughts (Hepburn, 2006, unpublished doctoral dissertation)

Comfort was assessed using 5 items. This scale was developed based on the idea that people might take refuge or comfort in suicidal thoughts or use them as a focus to regain mental control in times of distress. Participants were asked to consider their reactions to suicidal thoughts that came into their mind, and to rate how much they agreed with a series of items on a scale from 1=strongly disagree to 5=strongly agree. The items were ‘*suicidal thoughts come into my mind but do not bother me*’; ‘*I take comfort from thoughts of suicide*’; ‘*thinking about suicide makes me feel calm*’; ‘*thinking about suicide makes me feel better*’; and ‘*I think about suicide to help myself cope*’. The questionnaire was initially piloted on a group of 26 individuals with a history of suicidal ideation or behaviour participating in the control arm of a pilot randomised trial of Mindfulness Based Cognitive Therapy (Williams et al., 2008). Participants found the items comprehensible and the scale had good internal consistency, *α*=.81 (*n*=26).^2^ In the initial sample comfort correlated with worst ever suicidal ideation, *r* (16)=.49, *p*<.05. In the current sample the Cronbach's alpha for the comfort scale at the baseline assessment was *α*=.82 (*n*=217), again indicating satisfactory agreement (Bland and Altman, 1997). Participants completed the comfort scale at the baseline assessment, at the beginning of the follow-up phase – 8–12 weeks later and at approximately12-months follow-up. There was substantial stability in ratings of comfort from suicidal thoughts from baseline to the start of follow-up *r* (195)=.65, *ρ* (195)=.57 and 12 months follow-up assessments, *r* (172)=.61, *ρ* (172)=.51, all *p*<.001 indicating moderate to good test–retest reliability (Gwet, 2010).

#### 2.4.2 Beck Scale for Suicide Ideation: current (BSS) and worst ever (BSSw) versions (Beck and Steer, 1993)

The BSS assesses patients' current plans, thoughts and intent to die by suicide. It is a 21 items scale; the first 5 items are used as a screening tool and are completed by all individuals, irrespective of the severity of suicidal ideation. The remaining 16 items are completed only by those individuals who indicate that they have either a weak to moderate desire to kill themselves or that they would take a chance on life and death in a life threatening situation. For the purposes of the current paper analyses were based on these screening items, which provided a score ranging from 0 to 10. The Cronbach's alpha for the BSS screen in the current sample at the time of the baseline assessment was *α*=.89. Severity of suicidality during the follow-up phase was also based on the BSS screen score. The outcome variable was the highest BSS screen score from an assessment at which a relapse to major depression was recorded.

The BSSw questionnaire is a modified version of Beck Scale for Suicide Ideation based on the worst-point scale for suicide ideation interview (Beck et al., 1999). It asks patients about time during their lives when they have felt at their most down and depressed. Once the patient has identified this period they are asked to complete the same items as in the BSS, but worded in the past tense. The BSSw screen score (first 5 items) was used an indicator of worst-ever prior suicidality. The Cronbach's alpha for the BSSw screen score at the baseline assessment was *α*=.87.

#### 2.4.3 Beck Depression Inventory II (Beck et al., 1996)

Current symptoms of depression were measured using the Beck Depression Inventory (BDI-II) self-report questionnaire. The BDI-II contains 21 groups of statements that assess the presence and severity of depressive symptoms occurring within the preceding 2 weeks. Each item is ranked on a three points scale for a total score ranging from 0 to 63, with a score greater than or equal to 20 typically used to indicate at least moderate depression (Beck et al., 1996). The BDI-II is a well-established measure with high internal consistency. In the current sample the Cronbach's alpha for the BDI at baseline was *α*=.90.

#### 2.4.4 Life timeline measure

In order to assess lifetime history of depression, interviewers used a visual timeline with age depicted on the *x*-axis and level of depression on the *y*-axis. Auxiliary lines on the *x*-axis indicated the beginning and end of each year from age 10–70, auxiliary lines on the *y*-axis indicated the extreme points of worst and best mood and the zero point of the dimension. Participants were first asked to indicate on the timeline a number of anchor points that reflected important events or periods in their lives such as time they had lived in a particular city, the beginning and end of their school years, or further education, time they had been in a particular job, marriage, the birth of children or other events that individuals felt were important. They were then asked to mark the worst point of each episode of depression they had experienced. In a next step, participants connected the dots by indicating how levels of depression changed over time. Participants were asked to use the zero-point of the scale as a reference point indicating normal mood without any symptoms of depression and to mark changes in depression levels over time. Once participants had identified episodes of depression, they worked through the timeline indicating whether each episode had been accompanied by suicidal ideation or behaviour. From these timelines we derived four binary indices relating to clinical history; (i) whether or not suicidality was present in the first episode of depression, (ii) whether participants had been suicidal in more than one previous episode or not, (iii) whether first onset of suicidality occurred in adolescence or not, and (iv) whether first onset of depression occurred in adolescence. These data were available for participants at the Oxford site only.

### 2.5 Data analysis

Retrospective analyses were based on data from the *n*=217 individuals who reported previous suicidal ideation or behaviour at the baseline assessment and had available data on the comfort scale and BSSw (80% of total sample, 53 individuals did not report history of suicidality at baseline assessment, one had missing data on the baseline comfort scale and three had missing data on the BSSw). Prospective analyses were based on data from the *n*=98 individuals who had been followed-up until the point at which at least one relapse to depression was recorded and who had provided data on the BSS at one or more assessment at which a relapse had been recorded. There were no significant differences in age, gender, marital status, employment status, or baseline levels of depression (BDI-II) between those who contributed to prospective analyses and the remainder of the baseline sample. The mean duration of follow-up for these participants was 472 days (*SD*=76.79) and the mean time to first relapse was 167 days (*SD*=136.02). Where more than one relapse to depression was recorded the episode with the most severe suicidal ideation was selected for prospective analyses.

BSSw screen score did not differ significantly from normality but the comfort from suicidal thoughts scale and suicidal ideation at baseline and follow-up had substantial positive skewness. Transformation of the data did not render these variables normally distributed. We therefore used Spearman's non-parametric correlations to examine the association between ratings of comfort from suicidal thoughts at baseline assessment and severity of worst prior suicidal ideation for participants with a history of suicidal ideation or behaviour at the baseline assessment, and Mann–Whitney *U* tests to compare individuals with and without a history of suicidal behaviour in endorsement of comfort as well as analyses based on timeline data. A comparison of parametric and non-parametric correlations yielded materially equivalent results, so as no non-parametric equivalent to hierarchical linear regression exists, a hierarchical linear regression was used to explore the contribution of baseline comfort score to severity suicidal ideation at relapse during follow. Missing data handling is described in Appendix.

## 3 Results

### 3.1 Participant sociodemographic characteristics

The sample was 25% male (*n*=55), and 75% female (*n*=163) with a mean age of 42.65 years (*SD*=11.97). 95% of the sample was Caucasian. 65% were employed, either full-time or part-time, 21% were unemployed and the remainder were either retired, on sick leave or on disability benefits. 26% of the sample were single, 50% married or cohabiting and the remaining 24% were widowed, separated or divorced.

### 3.2 Comfort scale descriptives

**Fig. 1:**
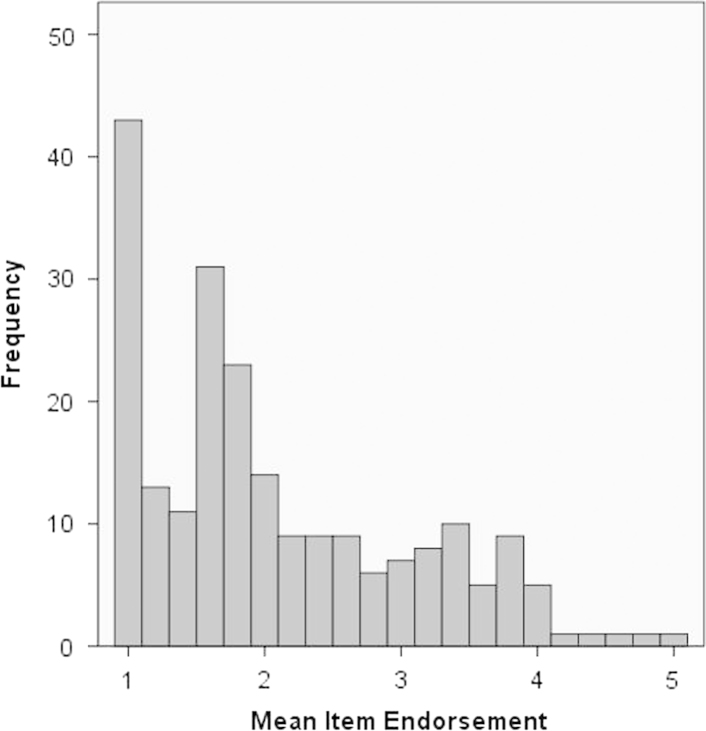
Mean item endorsement on the comfort scale at the baseline assessment (*n*=217). *Note*: mean item endorsement of 1 corresponds to an average rating of ‘strongly disagree’. Mean item rating of 3 corresponds to neither agree nor disagree. A mean item rating of 5 corresponds to ‘strongly agree’.

Mean item endorsement for the comfort scale at the baseline assessment is shown in Fig. 1. 26% of the sample had a mean item endorsement of 1 indicating that they strongly disagreed with all 5 comfort items and approximately 85% of the sample scored at or below the mid-point on the scale. This suggests that a majority of participants *do not* find suicidal ideation comforting. However the remaining 15% of the sample scored above the mid-point, indicating some degree of comfort in suicidal thoughts, and 15% of the sample strongly agreed with at least 1 item on the comfort scale. There was no significant difference between male (*M*=10.71, *SD*=5.12) and female (*M*=10.27, *SD*=4.76) participants in ratings of comfort from suicide, and no significant correlation between age and comfort from suicidal thoughts, *rho*=−.04, and =.50. There was also no significant correlation between baseline levels of depression (BDI-II) and comfort score, *rho*=−.09, and =.21.

### 3.3 Comfort scale and history of suicidality

The comfort score showed a modest but statistically significant correlation with worst ever suicidal ideation, Spearman's Rho (217)=.23, and *p*<.001, such that individuals who reported that they found their suicidal ideation more comforting, also reported more severe prior suicidal ideation on the BSS. However a Mann–Whitney *U* test indicated that there was no significant difference between individuals with a history of suicidal behaviour and those with a history of suicidal ideation (without behaviour) in comfort scores at the baseline assessment: ideation (*n*=136), *M*=10.82, *SD*=5.11, and versus behaviour (*n*=81): *M*=10.04, *SD*=4.69, *p*>.30.

### 3.4 Suicidal ideation during follow-up

A Spearman's rank order correlation was computed between comfort from suicide at the baseline assessment and severity of worst suicidal ideation at relapse during follow-up. This indicated a significant association, *ρ* (97)=.31, and *p*=.002. Future suicidal ideation is also predicted by past suicidal ideation, *ρ* (97)=.25, and *p*=.013 so in order to isolate the unique contribution of comfort from suicidal ideation to future suicidality a hierarchical linear regression analysis was conducted. This analysis examined the relationship between comfort from suicide and severity of suicidal ideation during follow-up, after taking into account suicidal ideation at baseline and severity of worst-ever suicidal ideation, two known predictors of future suicidality. Levels of suicidal ideation at the baseline assessment, and worst ever suicidal ideation (assessed at baseline) were entered at step 1 and comfort score was entered at step 2. At step 1 both worst ever BSS screen score, *B*=.301, *t*=3.42, and *p*=.001, and baseline current suicidal ideation, *B*=.418, *t*=4.76, and *p*<.001, entered as significant predictors of suicidal ideation during follow-up, resulting in a significant model, *F* (2, 94)=17.97, *p*<.001, and Adj *R*^2^=.26. The addition of comfort score at step 2 led to a significant improvement in model fit, Δ*F* (1, 93)=6.18, and *p*=.015, with comfort entering as contributing an additional 4.5% of the variance in severity of suicidal ideation.

### 3.5 Comfort score and suicidal behaviour during follow-up

Of the 217 individuals who had a history of suicidal ideation or behaviour at the baseline assessment 10 reported engaging in further deliberate self-harm during the follow-up period. There was no significant difference in reported comfort from suicidal thoughts between those who had an episode of self-harm than those who had not.

### 3.6 Clinical course and comfort from suicidal cognition

Data on clinical indicators derived from interview timelines were available for *n*=111 participants in Oxford. We used this data to compare comfort scores across groups defined according to various clinical characteristics identified on participants' timelines. These analyses indicated that there was a trend towards higher comfort scores in those who had been suicidal in their first episode of depression, Mann–Whitney *U* test *p*=.07, and significantly higher comfort scores in those participants who had been suicidal in more than one episode of depression, Mann–Whitney *U* test *p*=.046, and/or who had had an onset of depression in adolescence, Mann–Whitney *U* test *p*=.035. There was no significant difference in comfort scores between those with onset of suicidality in adolescence and those with onset of suicidality in adulthood, Mann–Whitney *U* test, *p*=.51.

## 4 Discussion

As well as considering the content and motivations for suicidal ideation and behaviour it is relevant to consider how people relate to their own suicidal cognitions. One recently emerging interest is in the degree of comfort associated with suicidal cognition, a factor noted across several studies exploring people's experiences of mental imagery at times of depressed mood and suicidal crisis (Holmes et al., 2007; Crane et al., 2012). The current study built on these findings to explore comfort from suicidal thoughts in people with a history of recurrent suicidal depression. Results showed that on average around 15% of the sample found suicidal thoughts non-aversive or comforting to some degree. This suggests that most individuals with a history of recurrent suicidal depression report little comfort from suicidal cognitions, but that such reactions and appraisals are present for a significant minority. The proportion of individuals reporting comfort from suicidal thoughts is somewhat lower than the proportions reporting comfort from suicidal imagery in previous studies. It is possible that there are differences between the degree of comfort associated with suicidal imagery and the degree of comfort associated with ‘suicidal thoughts’, which may have been interpreted by participants as referring primarily to thought content which is verbal in nature. It is a question for future research to address whether comfort is a property more closely associated with specific suicidal images than broader abstract verbal suicidal thoughts and/or whether some individuals experience some aspects of suicidal cognition as comforting whilst also experiencing other aspects as distressing.

Comfort from suicidal thoughts was associated in univariate analyses with more severe worst ever suicidal ideation, with having an onset of depression in adolescence, with being suicidal in more than one episode of depression, and at trend level, with being suicidal in the first episode of depression. These findings confirm the hypothesis that comfort from suicide is associated with a more severe clinical picture with the results showing that, as predicted, degree of comfort with suicidal cognition was associated with worse suicidal ideation during follow-up. Indeed although the absolute variance in suicidal ideation predicted by comfort from suicide was small (at around 4.5%), the fact that comfort has a significant association with future severity of suicidal ideation over and above worst ever suicidal ideation is noteworthy and potentially theoretically important. Although these findings cannot directly address the notion that individuals may habituate to suicidal cognitions across repeated episodes of suicidal ideation, similar to the habituation to suicidal behaviour described in Joiner's IPTS model (Van Orden et al., 2010), they would be broadly consistent with such a process and it would be valuable in future research to relate comfort from suicidal thoughts to the total lifetime duration of depression and the number of suicidal episodes an individual had experienced, to elucidate these issues further.

Interestingly, individuals who had previously engaged in suicidal behaviour did not report higher levels of comfort from suicidal thinking than those who had experienced suicidal ideation only. It is possible that episodes of suicidal behaviour and its aftermath actually reduce comfort from suicidal cognition in some circumstances, particularly if a suicide attempt has resulted in traumatic medical intervention, hospitalisation or distress in family and friends. Alternatively, because the questionnaire measure of comfort used focused specifically on suicidal thoughts, rather than suicidal acts, it may not have been sensitive to dimensions of appraisal relating to suicidal behaviour. Indeed O'Connor's (2011) integrated motivational–volitional model of suicidality suggests that the moderators that affect transitions to and intensity of suicidal ideation differ from those that affect enactment of suicidal behaviour, and it is possible that comfort, as measured here, relates only to the suicidal ideation component of the suicidal process.

Being, to the best of our knowledge, the first study to systematically assess comfort from suicidal cognition, this study has a number of limitations that should be borne in mind when considering the results. First, we assessed severity of suicidal ideation at times of relapse on five occasions during follow-up and took the highest score obtained. However some individuals may have experienced an escalation of suicidal ideation that was not captured by these discrete measurements. Clearly in future research it would be advantageous to explore the micro-processes through which an individual's response to emerging suicidal cognitions alters (or does not alter) the trajectory of cognitions over time and what the relationship is between comfort from suicidal thoughts and severity of depression or other psychopathology.

A second limitation is that our analysis is restricted to the study of the relationship between comfort from suicidal cognitions and reemergence of suicidal ideation, rather than suicidal behaviour. Although a significant proportion of the sample had engaged in suicidal behaviour in the past, rates of recurrence during the follow-up phase of the trial, in which all individuals were participating, were very low, and we did not have information on the intent of episodes of self-harm in the follow-up period. Although the low rate may in part reflect under-reporting, it may also reflect the fact that the measure of suicidal behaviour used was a lifetime measure and may not have been closely linked to proximal risk. In future studies it would be very interesting to extend this work to examine comfort from suicidal cognitions in populations with higher frequencies of suicidal behaviour.

Finally, although comfort from suicidal cognitions was significantly related to severity of ideation over the subsequent year, the absolute proportion of variance explained by comfort after accounting for clinical history was small. However, given that comfort is associated with severity of prior suicidality as well as future suicidality, and that habituation processes may occur, we would argue that it remains an important variable to consider clinically. It is not possible to alter a person's history of suicidal ideation or behaviour. However, it is possible to explore and work with their relationship with emerging suicidal cognitions, and to do so in a way that may reduce risk.

In conclusion, our data suggest that that it is possible to measure comfort from suicidal thinking with a high degree of consistency, using a self-report questionnaire. The measure showed moderate to good test–retest reliability over 1 year follow-up period, across recurrences of depression and new periods of suicidal ideation, and was meaningfully related to clinical outcomes. Although the results of this study clearly require replication in different clinical groups (for example where suicidality is comorbid with conditions other than recurrent depression) and in relation to different clinical outcomes (most notably suicidal and non-suicidal deliberate self-harm), we strongly encourage others to consider assessing comfort from suicidal cognition in their future work and to explore the usefulness of this concept clinically with their patients.

## Role of funding source

This study was funded by Grant GR067797 from the Wellcome Trust to J.M.G. Williams and I.T. Russell (Trial Registration no.: ISRCTN97185214). The funder played no role in study design or conduct, or the decision to submit this paper for publication. The authors have no conflicts of interest associated with the published work.

## Conflict of interest

The authors have no conflicts of interest associated with the published work.

## Appendix

Data was considered missing at random (MAR). Where less than or equal to 25% of items were missing for a participant on a given scale (or subscale, for scales with multiple factors), we used that participants mean of the available items as the scale or subscale score. Where more than 25% of the items were missing, but a participant had at least seven scores present for the time point in question from across 13 core outcome variables defined for the trial, missing values were replaced by a single imputed value based on the participant's responses to these other outcome variables assessed at the same time point, and age, gender, centre and treatment group. If values remained missing, but the participant had data for the measure in question for at least four of the six time points, scale scores were replaced with a single imputed value based on their scores on the measure at the other time points and age, gender, centre and group. Some participants still had missing values after the above method was used.

## Footnotes

2 Omission of the item ‘Suicidal thoughts come into my mind but do not bother me’ led to an improved alpha *α*=.91. However the main results were unchanged after removal of this item, and so we chose to retain the whole scale for the purposes of the current analysis.
